# Effect of Non-Thermal Atmospheric Plasma on Food-Borne Bacterial Pathogens on Ready-to Eat Foods: Morphological and Physico-Chemical Changes Occurring on the Cellular Envelopes

**DOI:** 10.3390/foods9121865

**Published:** 2020-12-14

**Authors:** Tamara Calvo, Miguel Prieto, Avelino Alvarez-Ordóñez, Mercedes López

**Affiliations:** Department of Food Hygiene and Technology and Institute of Food Science and Technology, University of León, 24071 León, Spain; tcalvc@gmail.com (T.C.); miguel.prieto@unileon.es (M.P.); aalvo@unileon.es (A.A.-O.)

**Keywords:** atmospheric plasma, food safety, ready-to-eat foods, cellular membrane damage

## Abstract

Currently, there is a need for new technological interventions to guarantee the microbiological safety of ready-to-eat (RTE) foods. Non-thermal atmospheric plasma (NTAP) has emerged as a promising strategy for inactivating microorganisms on thermo-sensitive foods, and the elucidation of its mechanisms of action will aid the rational optimization and industrial implementation of this technology for potential applications in the food industry. In this study, the effectiveness of NTAP for inactivating strains of *Salmonella* Enteritidis, *Salmonella* Typhimurium, *Escherichia coli* O157:H7 and *Listeria monocytogenes* contaminating the surface of different sliced RTE foods (“chorizo”, salami, bacon, smoked salmon, tofu and apple) was investigated. In addition, to further assess the bacterial inactivation mechanisms of NTAP, the morphological and physico-chemical damages in bacterial cells were analyzed. NTAP was effective for the surface decontamination of all products tested and, especially, of cut apple, where the microbial populations were reduced between 1.3 and 1.8 log units for the two *Salmonella* strains and *E. coli* O157: H7, respectively, after 15 min of exposure. In the rest of foods, no significant differences in the lethality obtained for the *E. coli* O157:H7 strain were observed, with inactivation rates of between 0.6 and 0.9 log cycles after a 15-min treatment. On the other hand, the strains from the rest of pathogenic microorganisms studied were extremely resistant on tofu, where barely 0.2–0.5 log units of inactivation were achieved after 15 min of plasma exposure. *S.* Enteritidis cells treated for 10 min exhibited noticeable morphological and structural changes, as observed by transmission electron microscopy, which were accompanied by a loss in membrane integrity, with an increased leakage of intracellular components and uptake of propidium iodide and marked changes in regions of their FTIR spectra indicating major alterations of the cell wall components. Overall, this indicates that loss of viability was likely caused for this microorganism by a significant damage in the cellular envelopes. However, the plasma-treated cells of *L. monocytogenes* did not show such obvious changes in morphology, and exhibited less marked effects on the integrity of their cytoplasmic membrane, what suggests that the death of this pathogenic microorganism upon NTAP exposure is more likely to occur as a consequence of damages in other cellular targets.

## 1. Introduction

Because of changes in consumers’ lifestyle, the demand for ready-to-eat (RTE) foods has increased considerably in the last few years. As these food products meet the specific needs of convenience, most of them are consumed without any culinary preparation to eliminate microbial loads. As a result, outbreaks of foodborne illnesses associated with the consumption of RTE foods have been recurrently reported [1]. Indeed, many studies have documented the presence of foodborne bacterial pathogens, including *Salmonella enterica* sevovar Enteritidis, *Salmonella enterica* sevovar Typhimurium, *Listeria monocytogenes* and verocytotoxigenic *Escherichia coli* in different RTE foods [2,3,4,5]. Therefore, there is a need for new technological interventions at the end of food processing in order to mitigate the risks associated with these products while also maintaining the organoleptic and other quality traits desired by consumers. Although several strategies are being explored for this purpose, Non-Thermal Atmospheric Plasma (NTAP) has been the focus of increased research in recent years due to the advantages it presents in comparison to the other microbial inactivation methods, such as its effectiveness at ambient temperature, lack of residual toxicity, low costs of operation and the possibility of food processing inside the package, among others [6,7,8]. Recent studies have shown that NTAP can reduce the microbiological risk of different RTE foods, including both raw and processed foods from animal and vegetable origin, while preserving their quality attributes [9,10,11,12,13].

NTAP is generated by subjecting a gas to an electric discharge under ambient temperature and atmospheric pressure conditions. This leads to the partial ionization of the gas molecules and the formation of large quantities of microbicidal active agents, such as excited and non-excited atoms and molecules, positive and negative ions, free radicals, electrons and ultraviolet radiation. Despite much research efforts, the main cellular targets and the underlying mechanisms of action of NTAP are far from clear. The contribution of ultraviolet (UV) radiation to the antimicrobial effect of NTAP obtained at atmospheric pressure is controversial, while most authors consider that reactive chemical species generated through gas ionization are responsible for the bacterial inactivation by causing severe damages on various cellular structures and macromolecules, including cellular envelopes, DNA and proteins [6,7,14,15,16,17,18,19,20,21]. The cell envelopes are under investigation as the primary target of NTAP treatments [22,23,24,25,26]. The plasma charged particles could cause mechanical damages to the cell envelopes, either through their physical impact [27] or their accumulation in certain parts of the cell surface resulting in cytoplasmic membrane electroporation [22]. However, reactive oxygen species and reactive nitrogen species are generally considered the NTAP key components that most contribute to the loss of cell viability, especially by oxidizing the polyunsaturated fatty acids of the cytoplasmic membrane [14,28], which are very susceptible to lipid peroxidation phenomena. In fact, it has been reported that lipid peroxidation can compromise the cell viability by modifying membrane properties, decreasing its permeability or even its integrity [6,14,17,19]. To the best of our knowledge, no publication so far has focused simultaneously on the effects of NTAP in the cell structure as shown by of Infrared spectra, electronic microscopy, UV spectrophotometry and flow cytometry.

The objective of this study was to evaluate the effectiveness of NTAP for the inactivation of *Salmonella enterica* serovar Enteritidis, *Salmonella enterica* serovar Typhimurium, *Escherichia coli* O157:H7 and *Listeria monocytogenes* on different sliced foodstuffs, including fermented sausages, such as “chorizo” and salami, bacon, smoked salmon, tofu and freshly cut apple, which might become contaminated with these pathogenic microorganisms through manipulation by food handlers and/or contaminated equipment including cutting boards, knives, and working surfaces. In addition, in order to gain insights into the mode of action of this non-thermal technology, the morphological and physico-chemical changes occurring on the cellular envelopes of these pathogenic microorganisms after NTAP treatments were also determined by monitoring the uptake of propidium iodide (PI), the loss of intracellular contents, the morphological alterations as revealed by transmission electron microscopy (TEM) analysis, and the global changes in cellular biochemical features as revealed by Fourier transform infrared (FTIR) spectroscopy.

## 2. Materials and Methods

### 2.1. Microorganisms and Culture Conditions

*Salmonella enterica* sevovar Enteritidis (CECT 4300), *Salmonella enterica* sevovar Typhimurium (CECT 443), *Escherichia coli* O157:H7 (ATCC 43895) and *Listeria monocytogenes* (ATCC 15313) were purchased from Colección Española de Cultivos Tipo (CECT, Spanish Type Culture Collection) or American Type Culture Collection (ATCC). Bacteria were revitalized in tubes containing 10 mL of Brain Heart Infusion broth (BHI, Oxoid, UK) at 37 °C for 24 h. These bacterial cultures were streaked onto BHI agar plates which, after their incubation under the same conditions, were stored at refrigeration temperature (4 ± 1 °C) as stock cultures. The experimental cultures were obtained by transferring a single colony from the stock cultures into a sterile tube with fresh BHI, which was incubated at 37 °C for 24 h, entering the stationary phase, with a final cell concentration of approximately 10^8^–10^9^ cfu/mL.

### 2.2. Preparation and Iinoculation of Samples

Commercial vacuum-packed slices of “chorizo”, salami, bacon and smoked salmon, as well as apples (“Fuji” variety) and heat-treated tofu (presented in orthogonal blocks and packed in an aqueous solution) were purchased in a local supermarket. These foods were selected due to their great variability in composition, especially in sodium chloride content, and physicochemical properties (pH, a_w_ and pH). Tofu and apples were cut to obtain slices of approximately 6 mm thick. Afterwards, slices of all products were cut into 20 mm diameter disks. Prior to inoculation of pathogenic bacteria, all food products, except apple and tofu samples, were subjected to a pre-decontamination step. For this purpose, sliced chorizo, salami, bacon and smoked salmon were pressurized (900 MPa for 5 min) in a model FPG 7100:9/2C Series Foodlab (Standsted Fluid Power Ltd., Essex, UK). After pressurization, the number of microorganisms detected on those samples were below 10 cfu/g, as this is our limit of detection of the methodology used. To inoculate food samples, from bacterial cultures, ten-fold serial dilutions were prepared for each bacterial strain in sterile 0.1% (*w*/*v*) peptone water (Oxoid). Then, 30 µL of the appropriate dilutions were inoculated onto the surface of foods in order to achieve a final concentration of 10^3^–10^4^ cfu per disk.

For the assessment of the cellular membrane integrity (PI uptake and measurement of intracellular leakage) and the morphological alterations occurring on the cellular envelopes of *S.* Enteritidis and *L. monocytogenes* (TEM analyses) after NTAP treatments, Whatman polycarbonate membrane filters of 0.2 µ pore retention and 25 mm diameter (Fisher Scientific, Loughborough, UK) were inoculated with each bacterial culture to achieve a cell density of ∼10^8^–10^9^ cfu/filter. After spreading out the inoculum on the entire upper surface of filters and food disks, samples were air-dried for 15 min in a laminar flow cabinet (Telstar, model BV-100, Bristol, PA, USA) before NTAP treatments were conducted.

### 2.3. NTAP Treatments

For the NTAP treatment, a specially designed lab-scale plasma jet (CP121 Plasma Demonstrator OMVE BV, Netherlands) was used following the methodology described by [29]. Further details regarding plasma diagnostics for an equivalent NTAP treatment unit can be found elsewhere [30,31]. The equipment operates at atmospheric pressure and at a temperature below 40 °C [32]. The 0 bias position (oxidoreduction conditions), the 2 mm hole nozzle, air as working gas at a flow rate of 10 L/min, and a voltage difference of 2 kV between electrodes were used. The equipment operated with an output power of 1 watt.

Inoculated food disks were exposed to NTAP at predetermined time intervals, for a period of up to 15 min, whereas inoculated membrane filters were plasma treated for 10 min (an exposure time which was previously shown to reduce the bacterial populations in ca. 1 log reduction. Once treatments were completed, cells were recovered from food disks and membrane filters by transferring them to sterile Falcon tubes containing either 10 mL of 0.1% peptone water or 10 mL of PBS (Merck, Darmstadt, Germany), respectively, and vortexing for 2 min. For the enumeration of survivors, aliquots of 1 mL were inoculated into petri dishes and 20 mL of molten BHI agar tempered at a temperature of about 45 °C was then added. Once solidified, these agar plates were incubated for 48 h at 37 °C and, subsequently, the number of colonies was counted. Longer incubation times did not increase the number of recovered survivors. The bactericidal effectiveness of NTAP was evaluated via the calculation of Log reductions, by using the formula below:

Log reduction = Log (N_control_/N_treated_)

where:

N_control_ = number of microorganisms in untreated control samples (CFU)
N_treated_ = number of microorganisms in NTAP-treated samples (CFU)

In all cases, three independent experiments were performed in different days, using a different bacterial culture in each trial. Also in the experimental set up were included controls, which consisted of inoculated food samples and membrane filters not exposed to NTAP. In each trial, one food sample and membrane filter were tested.

### 2.4. Fourier Transform Infrared (FTIR) Spectroscopic Analyses

The bacterial cultures obtained as described in Section 2.1 were centrifuged at 11,000 rpm for 20 min at 4 °C. The pellet was resuspended in 1 mL of Ringer solution, inoculating 50 μL on a window of Zn-Se (zinc-selenium), which was dried in an oven (30 min at 37 °C). Then the window was NTAP- treated for 10 min. In parallel, a control consisting of cells not subjected to the action of the plasma was prepared. These experiments were carried out in triplicate.

The infrared (IR) spectrum was obtained using a FT-IR spectrophotometer equipped with a MIRTGS detector (Perkin-Elmer System 2000 FT-IR, Whaltman, MA, USA). The measurements were collected in the wave number range of 3500 to 700 cm^−1^ with an interval of 1 cm^−1^. The spectral resolution was 4 cm^−1^. The final spectrum of the samples was obtained by averaging 20 scans. The digitalized IR spectra (comprising a total of 2800 points) were digitally stored and mathematically transformed, including normalization (0 for absorption at 1800 cm^−1^, 1 for maximum absorption, located around 1650 cm^−1^) and the second derivative (Savitzky-Golay algorithm). Afterwards, transformed spectra were recorded in ASCII format and, for calculation purposes, divided into five spectral windows: w_1_ (3000–2800 cm^−1^, influenced by functional groups of membrane fatty acids); w_2_ (1800-1500 cm^−1^, affected by amide I and amide II groups belonging to proteins and peptides); w_3_ (1500-1200 cm^−1^, mixed region influenced by proteins, fatty acids and phosphate-carrying compounds); w_4_ (1200–900 cm^−1^, providing information mostly for carbohydrates and polysaccharides in the cell wall); and w_5_ (900–700 cm^−1^, called “true fingerprint”, because of very specific spectral patterns) [33].

A reproducibility analysis of the triplicates of the IR measurements was performed. Reproducibility measurements (measuring the internal variability) were obtained averaging three independent measures of the Pearson correlation coefficient (between replicates 1–2, 2–3 and 1–3), according to the Equation (1), and this value was expressed as the Differentiation Index, *Di* [34].

(1) $$r_{y1y2}=\frac{\sum_{i=1}^{n}y1_{i}y2_{i}-n\bar{y1}\bar{y2}}{\sqrt{\sum_{i=1}^{n}y1_{i}^{2}-n{\bar{y1}}^{2}}\sqrt{\sum_{i=1}^{n}y2_{i}^{2}-n{\bar{y2}}^{2}}}$$

Where:

*y*1_i_, *y*2_i_: individual values of absorbance of the two spectra to be compared.

*n*: number of points in the spectrum range to be compared.

$\overset{´}{y1}$ & $\overset{´}{y2}$: arithmetic means values y1 and y2.

(ry1y2): value of the correlation coefficient. From this value the Differentiation Index Dy1y2 is defined, according to the Equation (2):

(2) $$D\cdot y1\cdot y2=\left(1-ry1\cdot y2\right)\cdot 1000$$

*Di* can have values between 0 and 2000. It will be zero when the values in the spectral ranges are identical; 1000 when the values represent an inverse correlation, and 2000 for spectra with negative correlation, i.e., uncorrelated spectra.

The calculation of *Di* was obtained for the full spectrum range (ranges 3000–2800 and 1800–700 cm^−1^) and independently for the five spectral windows described above. A hierarchical clustering analysis was performed on the IR spectra obtained, using the Pearson’s correlation coefficient as a measure of similarity between spectra. The final grouping was obtained using the Ward algorithm, checking different spectral windows. All calculations (including calculation of coefficients, grouping of variables, and graphs) were performed with the Statistica for Windows v. Program. 7 (Statsoft Inc., Tulsa, OK, USA).

### 2.5. Transmission Electron Microscopy (TEM) Analyses

Untreated and 10-min NTAP treated cells of *S*. Enteritidis and *L. monocytogenes* were harvested by centrifugation, fixed in 2.5% glutaraldehyde (TAAB Laboratories Ltd., Aldermaston, Berks, UK)-PBS for 3 h at 4 °C and washed three times with PBS. Cells were then fixed with osmium tetroxide (TAAB Laboratories)-1% PBS for 45 min at room temperature in the darkness. The fixed cells were washed again three times with PBS, pelleted in bacteriological agar (Oxoid, Hampshire, UK), dehydrated by passage through a graded series of etanol solutions and embedded in an epoxy resin (Epon 812; Tousimis, Rockville, MD, USA), which was polarized through its incubation for 48 h at 60 °C. Following this, ultrathin sections were collected onto a copper grid and stained with uranyl and lead. TEM micrographs were taken on at least ten different microscope fields using a JEOL 1010 microscope (JEOL Ltd., Tokio, Japan) at 80 kV.

### 2.6. Membrane Integrity Tests

#### 2.6.1. Assessment of Propidium Iodide (PI) Uptake

After recovering control untreated and NTAP-treated cells, bacterial suspensions were incubated with 1 µL of PI 1 mg/mL solution (Molecular Probes, Life Technologies, Grand Island, NY, USA) for 10 min at room temperature in the dark in order to allow for its internalization into the permeabilised cells. Afterwards, samples were centrifuged at 3220× *g* and 10 °C for 15 min in order to remove the excess PI. The supernatants were then discarded and the cellular pellets were resuspended in PBS. Flow cytometry experiments were carried out using a CyAn-adp flow cytometer (Beckman Coulter, Brea, CA, USA). Samples were excited using a 488-nm air-cooled argon-ion laser. The instrument was set up with the following configuration: forward scatter (FS), side scatter (SS) and red fluorescence (613/20 nm) for PI. The cell population was selected by manually gating in a FS *vs* SS dot plot, which allowed for the exclusion of aggregates and cell debris. Fluorescence histograms were represented in single-parameter histograms. Data were analysed with Summit version 3.1 software (Cytomation, Fort Collins, CO, USA).

#### 2.6.2. Measurement of the Leakage of Intracellular Nucleic Acids and Proteins

Bacterial suspensions (3-mL) prepared as described in Section 2.3 were filtered through a 25-mm-diameter, 0.22 µm pore size, Millex-GS syringe filter (Millex-GS, Millipore Co, Billerica, MA, USA) to remove bacteria. Then, the release of nucleic acids and proteins was estimated by measuring the absorbance of the filtrates at 260 and 280 nm, respectively, with a UV/Vis UV-3100PC spectrophotometer (WWR International, Radnor, Pennsylvania).

### 2.7. Statistical Analysis

The analysis of statistical differences in log reductions and optical density values was carried out through the analysis of variance (ANOVA-one way) after applying the Kolmogorov-Smirnov′s test with Lilliefors correction and Levene’s test to confirm that the experimental dataset followed a normal distribution and the variances were homogeneous, respectively. Statistically significant differences (*p* < 0.05) between means were detected using a post-hoc Tukey’s test. All statistical analyses were carried out using the statistical software IBM SPSS Statistics 21 (Armonk, NY, USA). 

## 3. Results and Discussion

### 3.1. Effectiveness of NTAP on Bacterial Inactivation

The effectiveness of NTAP for reducing *S.* Enteritidis, *S.* Typhimurium, *E. coli* O157:H7 and *L. monocytogenes* on “chorizo”, salami, bacon, smoked salmon, tofu and fresh-cut apple was evaluated after 4, 8 and 15 min of NTAP treatment. The results showed that this non-thermal technology was effective for the inactivation of all the studied foodborne pathogens (Table 1). However, the lethality achieved depended on the three variables tested, i.e., treatment time, type of food and bacterium assayed. Although in all cases an increase in exposure time led to more efficient bacterial inactivation, the increment of the lethality did not follow a first order inactivation kinetic, indicating that the remaining bacterial population may potentially became more tolerant to plasma. The exact nature of this phenomenon is poorly understood but it is frequently observed for various microorganisms after NTAP treatment, including *S*. Typhimurium [32], *E. coli* [35] and *L. monocytogenes* [29,36].

Overall, NTAP treatments were more effective for the microbial decontamination of apple samples, with 1.30, 1.34, 1.47 and 1.79 log reductions being achieved after a 15 min-treatment for *S.* Enteritidis, *S.* Typhimurium, *L. monocytogenes* and *E. coli* O157:H7, respectively. However, no significant differences (*p* < 0.05) in the lethal effects obtained on the rest of food products were observed for *E. coli*, reaching 0.30–0.41, 0.45–0.55 and 0.57-0.89 log reductions after NTAP treatments of 4, 8 and 15 min, respectively. A similar behaviour was observed for *L. monocytogenes* when treated for 4 min (0.29–0.37 log reductions) or 8 min (0.40–0.71 log reductions), whereas a reduced effectiveness of NTAP for the decontamination of tofu was found at the longest treatment time (15 min), with only 0.42 log cycles of inactivation being achieved on this foodstuff for *L. monocytogenes*. The lowest reduction in the population of both *S.* Typhimurium and *S.* Enteritidis was also obtained in tofu, with only 0.46 and 0.22 log reductions being detected, respectively, even with treatment times of up to 15 min. In addition, similarly to the findings for other species, no marked differences were found in the log reductions attained for each serovar of *Salmonella* among the different foods of animal origin. For example, in the case of *S.* Enteritidis, treatments of 4, 8 and 15 min caused 0.30–0.58, 0.51–0.77 and 0.82–1.09 log reductions, respectively.

A great variability in NTAP effectiveness against both vegetative cells and bacterial spores has been reported in the literature when identical treatments were applied onto different surfaces (microstructure) [27,36,37]. This suggests that some surface-associated factors, such as surface roughness, adsorption of diffusing plasma species, and moisture might affect the survival of microorganisms on food. For example, higher rates of inactivation have been described for seven bacterial species, including *S.* Enteritidis, *S.* Typhimurium, *L. monocytogenes* and *E. coli* O157:H7, on agar than on smoked salmon, suggesting the need to account for surface characteristics [36]. The effectiveness of NTAP as a food decontamination technique has been reported to vary among different foods. Thus, inactivation of *E. coli* was more effective on tomato than on lettuce [38] or strawberries [39], or on carrots than on apples [40]. A greater lethal effect was also observed on cheese slices than on ham [16], on sliced ham than on chicken breast fillets [41], or on tomato than on strawberries [39] for *L. monocytogenes*. Similarly, inactivation of *S.* Typhimurium on lettuce was greater than on strawberries and potato [37]. Overall, it is generally acknowledged that complex food surfaces, such as those with increased roughness and presence of irregularities, may provide numerous sites for microorganisms to fix and hide, thus decreasing the bactericidal effect of NTAP treatments. In fact, some studies have shown that inoculated microorganisms are capable of finding shelter within various food irregularities, such as cracks, grooves or gaps [37,39].

Of particular interest in our results is the fact that no clear differences in the rates of inactivation were observed between Gram-negative (*S.* Enteritidis, *S.* Typhimurium, *E. coli* O157:H7) and Gram-positive (*L. monocytogenes*) bacteria when identical NTAP treatments were applied on the different types of foods studied. Thus, although *S.* Enteritidis exhibited a significant (*p* < 0.05) higher resistance than *E. coli* and *L. monocytogenes* on tofu, minor or no significant inter-specific differences in plasma susceptibility were observed when NTAP treatments were applied to decontaminate slices of “chorizo”, salami, bacon and smoked salmon. For example, no significant differences among species (*p* < 0.05) were found in the log reductions achieved on bacon, regardless of the treatment time. Likewise, 8 and 15 min NTAP treatments caused similar log-reductions on salami, regardless of the bacterial species. Interestingly, despite the minor differences observed, *L. monocytogenes* was not generally more resistant to NTAP than Gram-negative bacteria. Gram-positive bacteria are generally considered to be more resistant to NTAP treatments than Gram-negative bacteria [39]. This is attributed to the presence of a thicker peptidoglycan layer on their cellular wall, which would provide a greater rigidity and resistance to the diffusion of plasma reactive species through the bacterial cell wall. However, some comparative studies testing the inactivation by NTAP of different bacterial species under identical treatment conditions have found similar resistance for particular species from both Gram-positive and Gram-negative groups [7,25,42,43]. There exist even some reports of strains of *L. innocua* [10], *L. monocytogenes* [44], *B. cereus* [45], *S. aureus* and *E. faecalis* [46] being more sensitive to NTAP than strains of *E. coli, S.* Typhimurium, *S*. Enteritidis, *Vibrio parahaemolyticus* and *Pseudomonas aeruginosa*, respectively. Results obtained in the present study could contribute to explain these apparently contradictory results.

The levels of inactivation obtained in the current study when NTAP treatments were applied on a range of RTE foods are probably not enough to assure, on its own, the safety of the products, especially when complete absence of some microorganisms, such as *Salmonella* spp., is seeked. Therefore, this non-thermal technology should be combined with other surface decontamination strategies in order to increase its antimicrobial effectiveness and achieve the desired microbial reductions. A recent study has described the potential of NTAP to be incorporated within a hurdles technology approaches [36]. An intelligent design of minimal processing strategies requires reliable knowledge on their mechanisms of action. Although recent investigations have tried to elucidate the mechanisms of microbial inactivation by different non-thermal plasmas obtained under atmospheric conditions, the specific mode of action is until date still unknown. Taking into account the large number of different reactive species present in plasma, it is considered that several structures and macromolecules in the cell are likely affected [17,18,21]. However, it has been speculated that the main target are the cellular envelopes [6,17,18,21,22,23,24,25,26]. In this study, several analytical techniques have been applied to characterize the morphological and physico-chemical changes occurring in bacterial cells after their exposure to NTAP for 10 min.

### 3.2. Bacterial Chemical Changes Induced by NTAP

A study of the infrared spectrum of control-untreated and 10 min-NTAP treated *S.* Enteritidis, *S.* Typhimurium, *E. coli* O157:H7 and *L. monocytogenes* cells was carried out using FT-IR spectroscopy. FT-IR spectroscopy is a physical-chemical method used to interpret the chemical composition of cells, reflecting the composition of cellular components (proteins, polysaccharides, fats and nucleic acids), and constitutes a suitable technique for the study of the molecular changes after exposure to stress [47,48,49]. The analysis of the IR spectra requires a previous processing that allows to minimize the methodological variability and amplify the spectral differences due to chemical variations. A mathematical transformation was carried out that allowed generating much more marked differences between the spectral characteristics of treated and untreated cells. The most important feature of this transformation is the use of derivations to reduce the variability due to the incubation and preparation of the samples.

**Figure 1:**
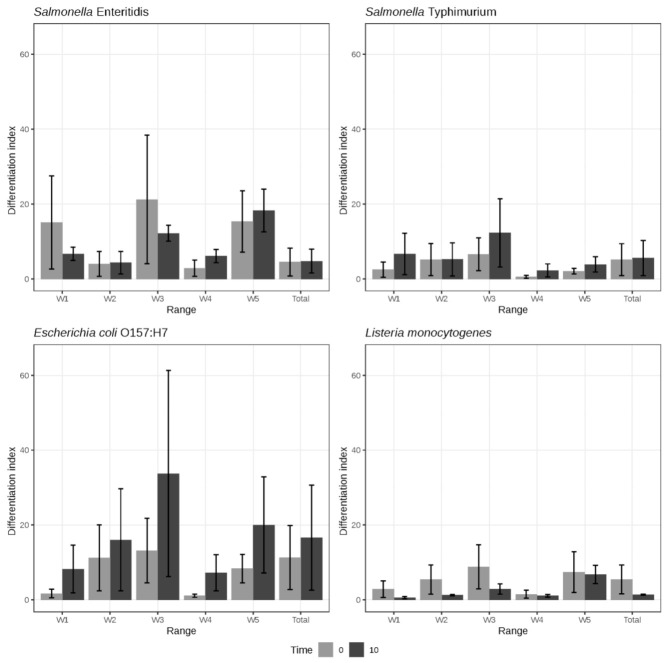
Differentiation index (Di) and global standard deviation calculated for *Salmonella* Enteritidis, *Salmonella* Typhimurium, *Escherichia coli* O157:H7, and *Listeria monocytogenes* for the full IR spectrum and for the five spectral windows.

The IR spectrum of the untreated cells for the four microorganisms studied (controls) was similar to IR spectra obtained in previous studies for *E. coli* O157:H7 [50], *S.* Typhimurium, *S.* Enteritidis [48]. The reproducibility was studied in detail for the five previously defined ranges (w_1_ to w_5_) and for the full spectrum. Three replicates per strain (NTAP-treated and controls) were processed in independent tests under the same experimental conditions. There are environmental factors that influence the reproducibility of the spectra (such as the manufacturing batch, the preparation of the culture medium, the temperature and the incubation time), as well as the conditions of measurement of the spectrum (the sample preparation, the calibration of the spectrometer and the conditions of measurement) [49]. To obtain classification schemes and perform correct identifications, the intra-replication variability must be minimized to the maximum, which is achieved using a careful standardization of the laboratory protocol and an adequate mathematical pre-treatment of the spectra.When the differentiation index (*D_I_*) is used to evaluate the reproducibility, global values between 7 and 10 are considered adequate when analyzing the replicates of samples prepared in independent trials, and the *D_I_* values can be up to 300 when comparing strains of different genera [49]. However, these values may vary depending on the types of microorganisms and the differences in chemical composition between them. Figure 1 presents the DI and the global standard deviation calculated for the four microorganisms: *S.* Enteritidis, *S.* Typhimurium, *E. coli* O157:H7 and *L. monocytogenes*, globally (complete IR spectrum) and for the five spectral windows.

**Figure 2:**
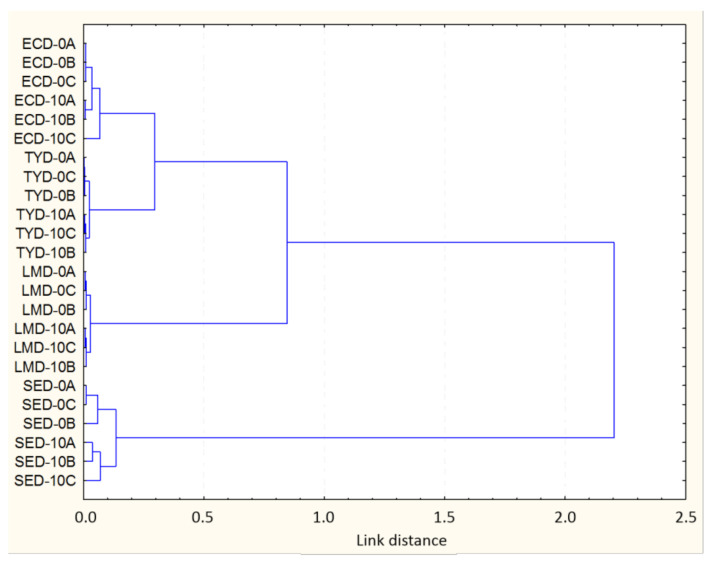
Clustering after the calculation of similarities (Pearson’s correlation coefficient) and cluster analysis (Ward method) of the window w_4_ of the IR spectrum of *Salmonella* Enteritidis (SED), *Salmonella* Typhimurium (TYD), *Escherichia coli* O157:H7 (ECD), and Listeria monocytogenes (LMD) after treatment by NTAP. The numbers indicate the treatment time (0 and 10 min) and the final letter (A, B, C), the replicate.

The results indicate that a good standardization of the conditions of incubation, treatment and processing of the sample, and of the spectroscopic analysis was obtained. Thus, according to the quality criteria for the *D_I_* index previously discussed [49], *S.* Enteritidis, *S.* Typhimurium and *L. monocytogenes* obtained adequate *D_I_* values (in the case of *S.* Enteritidis the appropriate values were only found in some windows, among them the w_4_ used in the cluster analysis, Figure 2). On the other hand, *D_I_* values were higher, indicating a lower reproducibility, for *E. coli* O157:H7. These results show that the *D_I_* value is a very reliable indicator of the quality of the spectra, since the results are similar to those found with the cluster analysis (Figure 2). Statistically significant differences (*p* < 0.05) were obtained between the different microorganisms. The replicas belonging to *L. monocytogenes* and *S.* Typhimurium offered the lowest *D_I_* values, both in the individual ranges and, logically, in the global measure. These data agree with the arrangement and grouping of the replicas observed in the cluster (Figure 2), which are described below. The w_4_ range generally obtained the lowest *D_I_* values for both processed and unprocessed samples (0.60 for S. Typhimurium, 1.13 for *E. coli* O157:H7, 1.53 for *L. monocytogenes*, 2.84 for *S.* Enteritidis). In contrast, windows w_3_ and w_5_ generally showed the highest *D_I_* values (33.74 for *E. coli* O157:H7 in w_3_ and 21.22 for *S.* Enteritidis). In the analysis of the *D_I_* values obtained for the NTAP-treated samples vs the control samples, NTAP-treated samples from *S.* Typhimurium and *E. coli* O157:H7 obtained greater variability compared to the untreated ones, in all the windows. However, for *L. monocytogenes*, control samples showed greater variability, and no clear trends were observed for *S.* Enteritidis.

The results show a great difference in the reproducibility observed between windows, which is attributed to changes in the chemical compounds of the sample that are reflected in the IR spectrum. Despite the standardization, the windows w_2_, w_3_ and w_5_ were the most variable. On the other hand, the window w_4_ showed a high degree of chemical stability as revealed by low *D_I_* values. These results agree with those previously obtained by us [34], in which values lower than 10 were obtained for the worst reproducibility window (w_5_) and lower than 1 for w_4_, with intermediate values of 4.58 and standard deviation of 3.05. The *D_I_* value obtained from the replicas can function as a quality parameter of the IR spectra allowing a priori knowledge of the quality and taxonomic or quimiotaxonomic significance of the subsequent cluster analysis. In this way, replicas that do not reach defined values would be discarded automatically.

To represent the existence of differences between strains treated and not treated by PANT, a study was carried out on the changes suffered in the IR spectrum. The hierarchical classification obtained using a cluster analysis of the second derivative of the IR spectra revealed differences between the treated and untreated cells, for the five spectral regions (w_1_–w_5_), although the w_4_ region, with a global distance of 2.3, was the most discriminating region (Figure 2). The results obtained for this spectral region showed that the conveniently transformed average IR spectrum is able to discriminate between intact (controls) and damaged cells. The resulting cluster presented four sub-clusters that grouped separately the strains belonging to each species, with taxonomic significance. Within each cluster, the strains were grouped separating the treated from the untreated (controls) samples. It should be noted that the global link distance was 2.3, intermediate value that indicates that the differences between clusters are adequate, although those present within each cluster are small and limited to small ranges of the window used (w_4_). These results agree with those obtained by [51] that show not only the potential of FT-IR spectroscopy to discriminate between intact and damaged cells, but also the ability of the technique to study the molecular aspects of the bacterial response to stress. Interestingly, while it was possible to discriminate treated from untreated cells for all the four microorganisms tested, the differences were more marked for Gram-negative bacteria, and especially for *E. coli* O157:H7 and *S.* Enteritidis, than for Gram-positive ones (*L. monocytogenes*) (Figure 2).

### 3.3. Bacterial Morphological Changes Induced by NTAP 

**Figure 3:**
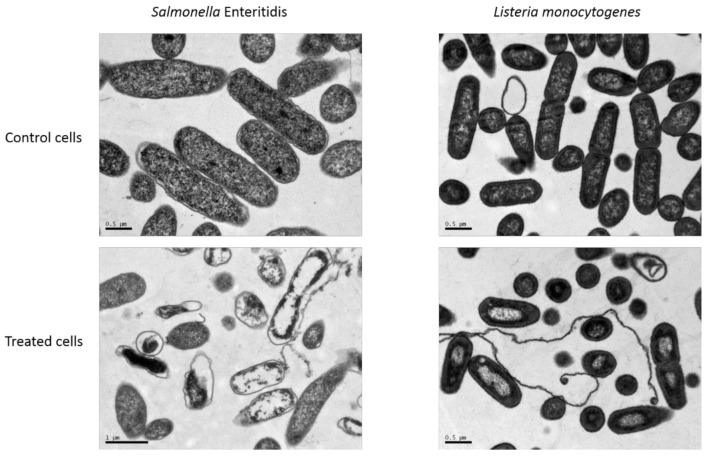
Representative transmission electron micrographs for cells of *Salmonella* Enteritidis and *Listeria monocytogenes* untreated and after NTAP treatment.

Figure 3 shows the morphology of cells of *S.* Enteritidis and *L. monocytogenes*, as representative of Gram negative and Gram positive bacteria, respectively, obtained from TEM analysis before and after a 10-min NTAP treatment. The untreated bacteria showed a typical spherical or elliptical shape, a smooth surface, and an intact cell wall and cell membrane. However, NTAP-treated cells of *S.* Enteritidis showed morphological and structural changes with notable shrinkage, deformation of the external shape, disruption or detachment from the cytoplasm of the cellular envelopes, and unevenness of the intracellular content. On the other hand, *L. monocytogenes* did not exhibit such obvious changes in morphology after NTAP treatment, although cells showed some blank spaces in their cytoplasm and condensation of the cytoplasmic material in amorphous compacted regions.

### 3.4. Damage in Bacterial Membrane Integrity Induced by NTAP

**Figure 4:**
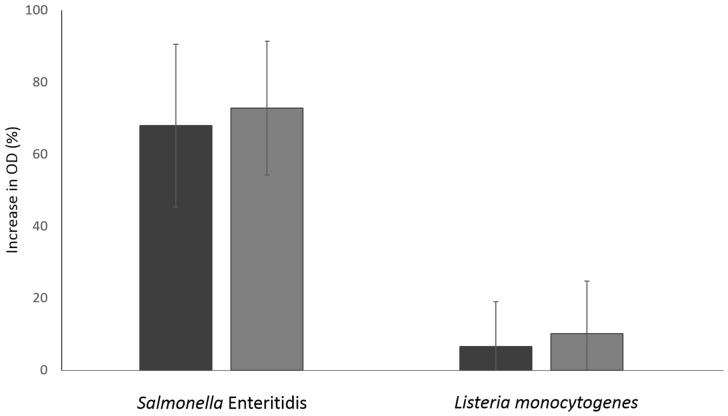
Optical density at 260 (black) and 280 (grey) nm of the cell-free filtrates of *Salmonella* Enteritidis and *Listeria monocytogenes* after their exposure to the action of NTAP. The graph represents the increase, expressed as percentage, in the optical density obtained for the treated cells in relation to that calculated for the untreated cells.

To further study the effects of NTAP, membrane integrity was assessed by measuring the UV absorbing intracellular components (i.e., the nucleic acids and proteins absorbing at 260 and 280 nm, respectively) of cell-free filtrates obtained from untreated and treated samples (Figure 4). While for *L. monocytogenes* no significant (*p* > 0.05) increases in the release of intracellular components were observed after exposure to NTAP, the treatment of *S.* Enteritidis cells markedly increased the content of both nucleic acids and proteins present in the cell-free filtrates in comparison with those obtained in the corresponding filtrates for the untreated control cells.

**Figure 5:**
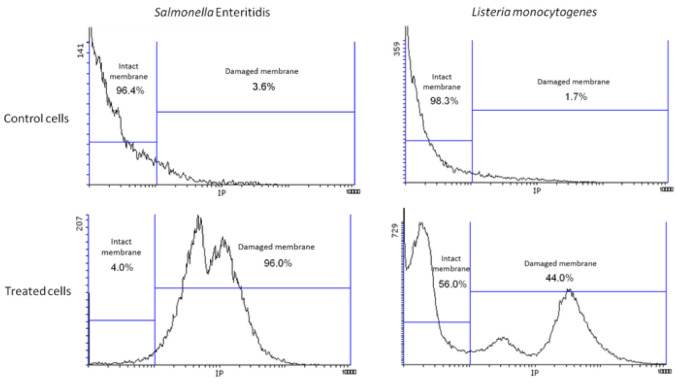
Flow cytometry histograms (red fluorescence) showing the uptake of propidium iodide (PI) by untreated and NTAP- treated cells of *Salmonella* Enteritidis and *Listeria monocytogenes*.

Membrane integrity was also assessed by measuring the intake of PI, a small fluorescent probe that binds to intracellular single- and double-stranded nucleic acids yielding red fluorescence, but cannot passively traverse into cells with intact cytoplasmic membranes [51]. For both *S.* Enteritidis and *L. monocytogenes*, less than 5% of untreated cells were PI stained (3.6% and 1.7%, respectively). However, after a 10-min NTAP treatment, whereas near 100% of *S.* Enteritidis cells (96%) were PI-positive, only a subpopulation of 44% of *L. monocytogenes* cells showed a damaged cytoplasmic membrane (Figure 5). Interestingly, the presence of this fraction of cells with an undamaged membrane correlates well with the concave upward survival curves previously observed for this pathogenic microorganism under identical experimental conditions [29]. It is noteworthy that the inactivation of *S.* Enteritidis followed, on the contrary, an exponential decay with time under the same experimental conditions [52].

Taking into account the results obtained for *S.* Enteritidis, i.e., the leakage of intracellular nucleic acids and proteins, the intake of PI, the visualization of broken cells and the higher discrimination between untreated and treated cells obtained for this bacterium in the w_4_ window in the FTIR spectroscopic analyses, it can be concluded that NTAP exerts its bactericidal effect in this pathogenic microorganism through the disruption of the cellular envelopes.

## 4. Conclusions

Results obtained confirm that NTAP is an effective technology in inactivating *S.* Enteritidis, *S.* Typhimurium, *E. coli* O157:H7 and *L. monocytogenes* on the surface of different RTE foods, reducing the microbial population up to 1.8 log units after a 15-min treatment. Although the cell membranes are considered the main target of NTAP, since they represent the first contact barrier with the reactive chemical species generated in plasma, the results obtained in the current study, through the application of different analytical techniques, suggest that the antimicrobial mechanism of action of NTAP could be different for Gram positive and Gram negative bacterial species as had been previously hypothesized [53]. Thus, the damages to the cell envelopes would be the main cause of viability loss for Gram negative bacteria, which would facilitate the action of some antimicrobial agents that, under normal conditions, are ineffective against this bacterial group due to their inability to pass through the outer membrane. This behavior opens the possibility of developing combined processes that would allow reducing the intensity of the treatments and/or increasing the antimicrobial effectiveness of NTAP, thus reducing its possible adverse effects on the quality attributes of foods. However, the inactivation of Gram positive bacteria is probably a consequence of the lesions caused in other cellular targets, such as the DNA and/or enzymes, given the ability of some of the chemical species present in plasma to cross the cell membranes and exert their action at intracellular level, as has been previously demonstrated [14,21,54]. All these aspects require a more in-depth study, since the knowledge on the molecular bases of the mechanisms involved in microbial inactivation would allow progress in the design of more effective treatments and particularly in the use of combined processes for food preservation.

## Figures and Tables

**Table 1:** Log cycles of inactivation obtained for *S.* Enteritidis, *S.* Typhimurium, *L. monocytogenes* and *E. coli* O157:H7 for different NTAP treatment times on different ready-to-eat foods.

Treatment Time	Food	Microorganisms			
		*S*. Enteritidis	*S*. Typhimurium	*E. coli* O157:H7	*L. monocytogenes*
4 min	“chorizo”	0.58 ± 0.05 _c_^1,2^	0.81 ± 0.10 _d_^2^	0.36 ± 0.07 _a_^1^	0.37 ± 0.13 _a_^1^
	salami	0.37 ± 0.01 _b_^1^	0.62 ± 0.13 _cd_^2^	0.32 ± 0.08 _a_^1^	0.32 ± 0.08 _a_^1^
	bacon	0.30 ± 0.04 _ab_^1^	0.34 ± 0.07 _a_^1^	0.33 ± 0.04 _a_^1^	0.38 ± 0.10 _a_^1^
	smoked salmon	0.40 ± 0.05 _bc_^1^	0.39 ± 0.06 _abc_^1^	0.30 ± 0.05 _a_^1^	0.29 ± 0.01 _a_^1^
	tofu	0.16 ± 0.06 _a_^1^	0.36 ± 0.04 _ab_^1^	0.41 ± 0.0 _a_^2^	0.34 ± 0.06 _a_^2^
	apple	0.59 ± 0.15 _c_^1^	0.60 ± 0.12 _bcd_^1^	1.31 ± 0.18 _b_^2^	0.89 ± 0.08 _b_^1^
8 min	“chorizo”	0.77 ± 0.13 _bc_^1,2^	1.03 ± 0.07 _d_^2^	0.55 ± 0.08 _a_^1^	0.71 ± 0.14 _a_^1^
	salami	0.58 ± 0.18 _b_^1^	0.75 ± 0.15 _bcd_^1^	0.48 ± 0.04 _a_^1^	0.45 ± 0.09 _a_^1^
	bacon	0.51 ± 0.09 _b_^1^	0.53 ± 0.15 _ab_^1^	0.48 ± 0.04 _a_^1^	0.56 ± 0.12 _a_^1^
	smoked salmon	0.70 ± 0.07 _bc_^2^	0.71 ± 0.09 _bc_^2^	0.45 ± 0.04 _a_^1^	0.49 ± 0.01 _a_^1^
	tofu	0.20 ± 0.07 _a_^1^	0.39 ± 0.04 _a_^2^	0.50 ± 0.07 _a_^2^	0.40 ± 0.06 _a_^2^
	apple	0.96 ± 0.10 _c_^1^	0.87 ± 0.11 _cd_^1^	1.50 ± 0.11 _b_^2^	1.12 ± 0.26 _b_^1,2^
15 min	“chorizo”	1.01 ± 0.10 _bc_^1^	1.20 ± 0.12 _b_^1^	0.89 ± 0.18 _a_^1^	1.04 ± 0.17 _bc_^1^
	salami	1.09 ± 0.10 _bc_^1^	1.10 ± 0.24 _b_^1^	0.65 ± 0.13 _a_^1^	0.80 ± 0.25 _ab_^1^
	bacon	0.82 ± 0.24 _b_^1^	1.00 ± 0.35 _ab_^1^	0.64 ± 0.14 _a_^1^	1.00 ± 0.20 _bc_^1^
	smoked salmon	0.86 ± 0.14 _b_^1,2^	1.02 ± 0.16 _b_^2^	0.57 ± 0.08 _a_^1^	0.57 ± 0.10 _ab_^1^
	Tofu	0.22 ± 0.03 _a_^1^	0.46 ± 0.03 _a_^2^	0.57 ± 0.06 _a_^2^	0.42 ± 0.10 _a_^2^
	apple	1.30 ± 0.20 _c_^1^	1.34 ± 0.11 _b_^1^	1.79 ± 0.06 _b_^2^	1.47 ± 0.19 _c_^1,2^

^a–^^d^ Mean values for each microorganism at the same treatment time with different subscript are significantly different (*p* < 0.05). ^1,2^ For each food, mean values with different superscript in the same row are significantly different (*p* < 0.05).
